# Axis I comorbidity in adolescent inpatients referred for treatment of substance use disorders

**DOI:** 10.1186/1753-2000-4-25

**Published:** 2010-09-28

**Authors:** Tobias Langenbach, Alexandra Spönlein, Eva Overfeld, Gaby Wiltfang, Niklas Quecke, Norbert Scherbaum, Peter Melchers, Johannes Hebebrand

**Affiliations:** 1 LVR Klinikum Essen - Kliniken/Institut der Universität Duisburg-Essen; Klinik für Psychiatrie und Psychotherapie des Kindes- und Jugendalters; Virchowstraße 174; 45147 Essen, Germany; 2 Kreiskrankenhaus Gummersbach - Klinik Marienheide; Leppestraße 65-67; 51709 Marienheide, Germany; 3 LVR Klinikum Essen - Kliniken/Institut der Universität Duisburg-Essen; Klinik für abhängiges Verhalten und Suchtmedizin; Virchowstraße 174; 45147 Essen, Germany

## Abstract

**Background:**

To assess comorbid DSM-IV-TR Axis I disorders in adolescent inpatients referred for treatment of substance use disorders.

**Methods:**

151 patients (mean age 16.95 years, SD = 1.76; range 13 - 22) were consecutively assessed with the Composite International Diagnostic Interview (CIDI) and standardized clinical questionnaires to assess mental disorders, symptom distress, psychosocial variables and detailed aspects of drug use. A consecutively referred subgroup of these 151 patients consisting of 65 underage patients (mean age 16.12, SD = 1.10; range 13 - 17) was additionally assessed with the modules for attention-deficit/hyperactivity disorder (ADHD) and conduct disorder (CD) using The Schedule for Affective Disorders and Schizophrenia for school-aged children (K-SADS-PL).

**Results:**

128 (84.8%) of the 151 patients were dependent on at least one substance, the remaining patients fulfilled diagnostic criteria for abuse only. 40.5% of the participants fulfilled criteria for at least one comorbid present Axis I disorder other than substance use disorders (67.7% in the subgroup additionally interviewed with the K-SADS-PL). High prevalences of present mood disorder (19.2%), somatoform disorders (9.3%), and anxiety disorders (22.5%) were found. The 37 female participants showed a significantly higher risk for lifetime comorbid disorders; the gender difference was significantly pronounced for anxiety and somatoform disorders. Data from the underage subgroup revealed a high prevalence for present CD (41.5%). 33% of the 106 patients (total group) who were within the mandatory school age had not attended school for at least a two-month period prior to admission. In addition, 51.4% had been temporarily expelled from school at least once.

**Conclusions:**

The present data validates previous findings of high psychiatric comorbidity in adolescent patients with substance use disorders. The high rates of school refusal and conduct disorder indicate the severity of psychosocial impairment.

## Background

The misuse of psychotropic substances is one of the most prevalent mental disorders in industrial nations and drug use is a frequent problem therapists in both adolescent and adult psychiatric settings must deal with. Johnston et al. [1] stated that 47% of all US-American adolescents have tried an illicit drug by the time they finish high school with cannabis being the predominant illicit drug. Estimated lifetime prevalences of substance use disorders (SUD) in adolescence range from 4.6% [2] to 12.3% [3]. Treatment research on both clinically ascertained adult substance-users [4] and on drug users in the adult general population [5,6] emphasise the basic negative influence of comorbid psychopathology on the outcome of drug-specific treatment, abstinence and rate of relapse. While a few community studies on adolescent drug use and their link to comorbid disorders and psychosocial problems have been conducted [6-11], only single studies examined the concurrent occurrence of SUD and other axis-I disorders on adolescent drug abusers seeking specific drug treatment [12,13]. Whereas epidemiological studies of the general population have often assessed all common axis-I diagnoses, the majority of studies concerning adolescent SUD and psychiatric comorbidity focused on selected comorbid mental disorders (ADHD and CD: [14-17]; anxiety disorders and depression: [18]; psychosis: [19,20]; various disorders: [21-26] or presented data based on broad diagnostic categories ("internalizing - externalizing" [27], "affective disorders - anxiety disorders" [28]). To our knowledge, only three recent studies on adolescent SUD-inpatients presented comprehensive data on the most common DSM axis-I disorders using standardized clinical interviews [29-31]. Only Kelly et al. [31] assessed comorbidity according to DSM-IV [32] whereas Jainchill et al. [30] and Hovens et al. [29] used DSM-III-R criteria [33].

Reflecting the health care system in many countries, most studies were conducted on outpatients or patients in residential programs. As a result, there is insufficient knowledge about psychiatric comorbidity in adolescent inpatients. As far as we know, only Deas et al. [28] and Hovens et al. [29] conducted their studies on inpatients, whereas other studies focused on outpatients or residential patients or considered inpatients within a heterogeneous group of inpatient, outpatient and residential patients [22,31].

To evaluate the temporal stability and developmental pathways of comorbid mental disorders, data on both current and lifetime comorbidity are required. However, to our knowledge, all recent studies limit the timeframe to either current or lifetime disorders. Furthermore, even the rates of current disorders are not based on the same timeframe; 12-month-, six-month and point prevalences of disorders are accepted indices to describe rates of present morbidity.

In light of the aforementioned limitations it should be noted that adolescent SUD patients very often suffer from externalizing disorders (Oppositional defiant disorder, CD, ADHD) and to a somewhat lesser extent from anxiety and mood disorders. Based on ten recent studies, Couwenbergh et al. [13] computed weighted means for the most relevant disorders: Mood disorders (26%), anxiety disorders (7%), PTSD (11%), ADHD (22%), CD (64%), and any comorbid mental disorder (74%).

Little research has been conducted on the consequences of maladaptive substance use concerning, school refusal and the link to comorbid mental disorders. Although some researchers [22,27,29] describe aspects of school attendance, there is still a lack of information about this important parameter of social functioning.

Psychiatric SUD treatment of adolescent inpatients differs in various ways from SUD treatment or detoxification of adults. Many practitioners agree that inpatient adolescent SUD treatment far more often has to account for specific difficulties like inactivity, high rates of treatment dropout and oppositional disorders. In many cases it remains unclear whether these problems are part of an age-appropriate developmental process or symptoms of a mental disorder. In the case of a comorbid axis-I disorder, misinterpreting these symptoms as normal adolescent-like behaviour or part of the substance use disorder would possibly delay the treatment of the comorbid disorder for a considerable time.

Although some practice-oriented treatment programs have been developed in the last decade many therapy concepts focus on consumption-related symptoms of SUD like withdrawal or maintenance of abstinence. Relating to the detoxification of adults or outpatient treatment of moderate SUD, this priority may be a reasonable approach. In the area of inpatient SUD treatment of adolescents this procedure runs the risk of neglecting severe psychosocial symptoms like school refusal or evolving delinquent/aggressive behaviour. This present study aims to provide further comprehensive data on psychiatric comorbidity of adolescents with substance use disorders with an additional focus on both gender aspects and school refusal. Furthermore we address some developmental psychopathological data as we include both lifetime and present axis-I diagnoses considering the changes in psychopathology.

## Methods

### Participants

Participants were 151 (114 male, 37 female) adolescents and young adults (≤22 years) referred for inpatient substance abuse treatment between April 2005 and December 2006. Patients were consecutively recruited in SUD-treatment units of the Rheinische Kliniken Essen (99 patients) and Kreiskrankenhaus Gummersbach - Klinik Marienheide (52 patients). Both units are located within child and adolescent psychiatric departments, providing full-service psychiatric health care. The Rheinische Kliniken Essen is situated in a metropolitan area of Germany whereas the Klinik Marienheide is located in a rural region. The procedure of admission to both drug-specific inpatient programs was comparable; in both units patients were required to be heavy drug users with clinically significant impairment or distress. Inclusion criteria were at least one SUD (other than tobacco-SUD) according to DSM-IV-TR [34], age between 12 and 22 years and inpatient treatment for at least two weeks. Patients were only excluded from the study if they were suffering from a severe acute psychotic disorder or a comparable condition, thus unable to participate in a clinical interview (n = 2). Study participation was strictly voluntary and signed informed consent was obtained from all participants and (in the case of minors) their parents/guardians. The participants and their parents/guardians had been informed about the study both orally and in written form. Only eight patients refused to participate. None of the remaining participants withdrew their participation. The mean age of the participants was 16.95 years (SD = 1.76), ranging from 13 to 22. The two study groups did not differ significantly in age (t = .996, p = .321) or gender (phi = .106, p = .233). Detailed site comparisons can be found in table 1. In the two weeks prior to admission, 34.5% of all participants lived together with their parents, 19.6% with a single parent, 18.9% in youth welfare service homes or residential programs for drug abusing adolescents and 18.2% of the subjects lived on their own (sometimes supported by social workers) or together with their partner or friends; 4.1% lived together with relatives or in a foster family, and 4.7% of the participants defined their life situation prior to admission as "miscellaneous", most often including short term homelessness. The study was approved by the Ethics committee of the University Duisburg-Essen.

**Table 1:** Site comparison

	Site 1 (Essen) n = 99		Site 2 (Gummersbach) n = 52	
	
	Present (%)	Lifetime (%)	Present (%)	Lifetime (%)
Gender				
male	78 (78.8)	-	36 (69.2)	-
Age (Mean)	17.95 (SD = 1.84)	-	16.75 (SD = 1.61)	-
SUD^a^				
Alcohol	36 (36.4)	44 (44.4)	13 (25.0)	14 (26.9)*
Cannabis	80 (80.8)	86 (86.9)	38 (73.1)	42 (80.8)
Amphetamine-like substances	22 (22.2)	28 (28.3)	5 (9.6)	6 (11.5)*
Hallucinogens^b^	7 (7.1)	11 (11.1)	0 (0.0)	1 (1.9)*
Cocaine	8 (8.1)	9 (9.1)	0 (0.0)*	1 (1.9)
Opiates	10 (10.1)	10 (10.1)	1 (1.9)	1 (1.9)
Inhalants	1 (1.0)	2 (2.0)	1 (1.9)	1 (1.9)
Sedative	2 (2.0)	4 (4.0)	0 (0.0)	2 (3.8)
Polysubstance	0 (0.0)	1 (1.0)	13 (25.0)***	15 (28.8)***
				
*Mood disorders*	21 (21.2)	23 (23.2)	8 (15.4)	10 (19.2)
*Anxiety disorders*	17 (17.2)	21 (21.2)	17 (32.7)*	19 (36.5)*
Adjustment disorder	0 (0.0)	0 (0.0)	2 (3.8)*	2 (3.8)*
*Somatoform disorders*	8 (8.1)	14 (14.1)	6 (11.5)	8 (15.4)
ADHD^c^	3 (12.0)	8 (32.0)	3 (7.5)	5 (12.5)
Conduct disorder^c^	12 (48.0)	19 (76.0)	15 (37.5)	20 (50.0)*
				
**Axis I disorder(s)^d^**	36 (36.4)	41 (41.4)	25 (48.1)	28 (53.8)

*Note: ^a ^*SUD = Substance use disorder: abuse or dependence according to DSM-IV-TR, without nicotine SUD. ^b ^including psychotropic mushrooms. ^c ^Subgroup, n = 65. ^d ^without CD and ADHD. * p < .05, ** p < .01, *** p < .00.

### Measures

During the second or third week of inpatient treatment, independent face-to-face interviews and questionnaires were conducted with the subjects. All interviews and questionnaires were administered by trained medical students or graduated, experienced clinical psychologists. One experienced clinical psychologist for each hospital acted as supervisor and guided the examiners. The clinical examinations lasted three and a half hours on average and were composed of six modules.

(1) The German edition [35,36] of the Composite International Diagnostic Interview (CIDI) [37]. This computerized interview measures DSM-IV Axis I disorders including substance-related disorders, mood, psychotic, anxiety, adjustment, somatoform and eating disorders.

(2) To access the DSM-IV-TR disorders ADHD and conduct disorder (DSM-IV-TR code 312.8), which are not included in the CIDI, the corresponding modules of the Schedule for Affective Disorders and schizophrenia for school-aged children - Present and Lifetime Version - German version (K-SADS-PL, Version 1.0) [38-40] were additionally administered consecutively to a limited subgroup (n = 65) of underage (< 18 years) participants. A present diagnosis represents a disorder that fulfils the respective DSM-IV-TR criteria during the last six months, lifetime diagnosis includes any diagnosis that appeared during lifetime, including present disorders

(3) The Fagerström Test for Nicotine Dependence (FTND) [41] was used to rate the extent of nicotine-addiction on a dimensional scale.

(4) The German version [42] of the Symptom Checklist-90-R (SCL-90-R) [43] evaluates a broad range of psychological problems and symptoms of psychopathology. Due to the high degree of reading difficulties apparent in the patients, this questionnaire was additionally orally explained by the investigators.

(5) Detailed information about drug-consumption for all relevant substances (e.g. onset of drug-use, present substance use, consumption in the last 30 days) obtained by comprehensive semi-structured interviews was recorded.

(6) A semi-structured interview slightly modified according to the Adolescent Drug Abuse Diagnosis (ADAD) [44] was used to collect data about school attendance, life situation and state of health.

Due to the fact that some parents of the participants either did not cooperate in a required manner or had no contact to their children for a long time, all interviews and questionnaires were carried out with the patients only.

### Statistical Analyses

Means, standard deviations and percentages were calculated to describe aspects of drug-use. To study possible differences between groups, the phi coefficient was used to examine nominal data, Student's t-test for interval and ANOVA for comparisons of interval data with more than two groups. Tests of significance were two-tailed using exact tests procedure for nonparametric statistics. The level of statistical significance was set at p < .05. Missing data (in five cases) have been substituted by the mean of the respective variable. All statistical analyses were carried out using SPSS V14.0.

## Results

### Substance use

Tobacco (99.3%), cannabis (84.8%) and alcohol (64.9%) were the most commonly used substances as well as the substances most often associated with SUD (table 2). Regarding present dependence on illicit drugs, nearly half of the patients were dependent on cannabis only (table 3). Patients who fulfil criteria for a present alcohol or cannabis dependence used these substances for a significantly longer time than patients without present dependence (table 4). With regard to nicotine dependence (measured with the FTND), the mean score of 5.18 (SD = 2.16) was in the range of a medium nicotine dependence. 13.2% of the patients were rated as having a very low level of nicotine dependence, 17.2% as having low dependence, 20.5% medium, 39.1% high and 9.9% as having a very high nicotine dependence.

**Table 2:** Substance use and substance use disorders

	Consume* (%)		Age of first use (SD)	Days of use*^# ^(SD)	Present disorder^+ ^(%)			Lifetime disorder (%)		
					
	present	lifetime			abuse	dependence	SUD	abuse	dependence	SUD
Tobacco	150 (99.3)	151 (100)	11.57 (2.21)	29.67 (2.32)	-	-	-	-	-	-
Alcohol	98 (64.9)	145 (96,0)	12.97 (1.73)	8.80 (7.74)	29 (19.2)	20 (13.2)	49 (32.5)	42 (27.8)	29 (19.2)	58 (38.4)
Cannabis	128 (84.8)	150 (99.3)	13.22 (1.46)	18.57 (9.10)	17 (11.3)	101 (66.9)	118 (78.1)	39 (25.8)	106 (70.2)	128 (84.8)
Ecstasy	33 (21.9)	88 (58.3)	15.24 (1.46)	5.87 (5.12)	8 (5.3)	19 (12.6)	27 (17.9)	15 (9.9)	22 (14.6)	34 (22.5)
Amphetamine	54 (35.8)	102 (67.5)	15.30 (1.44)	10.50 (9.05)	8 (5.3)	19 (12.6)	27 (17.9)	15 (9.9)	22 (14.6)	34 (22.5)
Hallucinogens^a^	13 (8.6)	66 (43.7)	15.69 (1.31)	2.83 (2.82)	3 (2.0)	4 (2.6)	7 (4.6)	6 (4.0)	6 (4.0)	12 (7.9)
Cocaine	13 (8.6)	58 (38.4)	16.09 (1.72)	8.31 (7.42)	1 (.7)	7 (4.6)	8 (5.3)	2 (1.3)	8 (5.3)	10 (6.6)
Opiates	6 (4.0)	23 (15.2)	15.26 (1.84)	26.17 (6.15)	4 (2.6)	7 (4.6)	11 (7.3)	6 (4.0)	8 (5.3)	11 (7.3)
Inhalants	7 (4.6)	34 (22.5)	14.76 (2.13)	12.57 (10.33)	0 (0)	2 (1.3)	2 (1.3)	1 (.7)	2 (1.3)	3 (2.0)
Polysubstance	-	-	-	-	2 (1.3)	11 (7.3)	13 (8.6)	3 (2.0)	13 (8.6)	16 (10.6)

*consume in the last 30 days, ^#^calculated for those patients with present consumption, ^+^criteria fulfilled for the last six months, ^a ^including psychotropic mushrooms; all percentages are based on the total study group of n = 151.

**Table 3:** Present substance dependence (excluding nicotine dependence)

Substance	**present dependence **(%)
Cannabis only	69 (45.7%)
Polysubstance use	11 (7.3%)
Cannabis and amphetamine-like substances	10 (6.6%)
Alcohol only	9 (6.0%)
Cannabis and alcohol	7 (4.6%)
other single substances, dependence rates < = 2%	5 (3.3%)
other substance use combinations	17 (11.3%)
No present dependence	23 (15.2)

**Table 4:** Duration of substance use in years in relationship to both dependency and comorbidity

	Substance use (years)					
	Dependence* Mean (SD)	No dependence* Mean (SD)	**Nominal p**	**Comorbidity^+^** **Mean (SD)**	**No comorbidity^+^** **Mean (SD)**	**Nominal p**

Alcohol	5.05 (2.48)	3.85 (1.91)	.018	4.19 (1.85)	3.90 (2.15)	.387
Cannabis	4.11 (1.89)	2.96 (1.50)	.001	3.91 (1.89)	3.61 (1.82)	.329
Amphetamine	2.42 (1.90)	1.78 (1.38)	.098	1.82 (1.49)	1.95 (1.52)	.665
Ecstasy	2.73 (2.05)	1.89 (1.41)	.063	1.94 (1.63)	2.09 (1.52)	.654

Note: *referred to the corresponding substance, *present *diagnoses. ^+^*Present *axis-I disorders excluding ADHD and CD.

### Prevalence of comorbid mental disorders

Dysthymic disorders, posttraumatic stress disorder and anxiety disorders in general were commonly found as comorbid diagnoses (table 5). Moreover, the patients who were additionally interviewed with the K-SADS revealed high rates of present CD and even higher lifetime rates of CD seemingly indicating that a notable proportion (30.8%) of lifetime CDs had remitted at time of admission. An analysis of links between ADHD and CD showed that 84% of the participants with a lifetime diagnosis of ADHD also had a lifetime diagnosis of CD (phi = .251, p = .059). Moreover, patients with one or more lifetime comorbid mood disorders (entire sample) tended to be older (17.52, SD = 1.92 vs. 16.81, SD = 1.70; T = -1.96, p = .052) than patients without a mood disorder. No relevant relationship between age and number of comorbid diagnoses (table 6) was detectable. With one exception (*present *somatoform disorders), no statistical relationship between age and specific comorbid axis-I disorders could be found (table 7).

**Table 5:** Comorbid DSM-IV-TR diagnoses

	Total (n = 151) Age = 16.95 (1.76)		Subgroup (with K-SADS) (n = 65)^a ^Age = 16.12 (1.10)	
	**Present (%)**	**Lifetime (%)**	**Present (%)**	**Lifetime (%)**

*Mood disorder*	29 (19.2)	*33 (21.9)*	*12 (18.5)*	*13 (20.0)*
Major depressive episode	5 (3.3)	7 (4.6)	3 (4.6)	4 (6.2)
Dysthymic disorder	24 (15.9)	24 (15.9)	8 (12.3)	8 (12.3)
Bipolar disorders	3 (2.0)	6 (4.0)	2 (3.1)	3 (4.6)
*Anxiety disorder*	34 (22.5)	*40 (26.5)*	*19 (29.2)*	*22 (33.8)*
Panic disorder with agoraphobia	4 (2.6)	5 (3.3)	2 (3.1)	3 (4.6)
Panic disorder w/o agoraphobia	3 (2.0)	3 (2.0)	0 (0.0)	0 (0.0)
Specific phobia	10 (6.6)	13 (8.6)	3 (4.6)	4 (6.2)
Social phobia	2 (1.3)	4 (2.6)	1 (1.5)	3 (4.6)
Obsessive-compulsive disorder	2 (1.3)	2 (1.3)	2 (3.1)	2 (3.1)
Posttraumatic stress disorder	12 (7.9)	12 (7.9)	9 (13.8)	9 (13.8)
Generalized anxiety disorder	3 (2.0)	4 (2.6)	1 (1.5)	1 (1.5)
Anxiety disorder NOS	3 (2.0)	3 (2.0)	3 (4.6)	3 (4.6)
Adjustment disorder	2 (1.3)	2 (1.3)	2 (3.1)	2 (3.1)
*Somatoform disorders*	14 (9.3)	22 (14.6)	8 (12.3)	13 (20.0)
*Eating disorders*	0 (0)	0 (0)	0 (0)	0 (0)
				
ADHD	-	-	6 (9.2)	13 (20.0)
Conduct disorder	-	-	27 (41.5)	39 (60.0)
				
	
**Axis I disorder(s)**	61 (40.5)*	69 (45.7)*	**44 (67.7)**	**53 (81.5)**

*Note: *ADHD = Attention-Deficit/Hyperactivity Disorder; CD = Conduct disorder; NOS = Not otherwise specified.

^a ^only participants 17 years old or younger. *without CD and ADHD.

**Table 6:** Number of comorbid DSM-IV-TR diagnoses (without SUD)

	Total (n = 151) Age = 16.95 (1.76)		Subgroup (with K-SADS) (n = 65)^a ^Age = 16.12 (1.10)	
	
	Present (%)	Lifetime (%)	Present (%)	Lifetime (%)
*Mean number of diagnoses (SD)*	*.58 (SD .89)*	*.71 (SD 1.00)*	*1.18 (SD 1.10)*	*1.65 (SD 1.22)*
0	90 (59.6)	82 (54.3)	21 (32.3)	12 (18.5)
1	43 (28.5)	44 (29.1)	23 (35.4)	22 (33.8)
2	14 (9.3)	17 (11.3)	10 (15.4)	13 (20.0)
3	2 (1.3)	5 (3.3)	10 (15.4)	13 (20.0)
4	1 (.7)	2 (1.3)	1 (1.5)	5 (7.7)
5	0 (0)	0 (0)	0 (0)	0 (0)
6	1 (.7)	1 (.7)	0 (0)	0 (0)

**Table 7:** Correlation between age and comorbidity

	Mean Age (SD)		t	p
	Present comorbidity	No present comorbidity		
*Mood disorders*	17.52 (1.92)	16.81 (1.70)	-1.96	.052
*Anxiety disorders*	16.85 (1.46)	16.97 (1.85)	.35	.725
Adjustment disorder	16.50 (.71)	16.95 (1.77)	.36	.719
*Somatoform disorders*	16.29 (.73)	17.01 (1.82)	2.93	.006
ADHD^a^	16.50 (.55)	16.08 (1.13)	-.88	.381
Conduct disorder^a^	16.11 (1.01)	16.13 (1.17)	.07	.942
				
**Axis I disorder(s)^b^**	17.00 (1.65)	16.91 (1.84)	-.30	.762

*Note: *^a ^Subgroup, n = 65. ^b ^without CD and ADHD.

### Psychological variables

Results from the symptom-checklist SCL-90-R revealed no statistically significantly elevated symptom distress in our sample in comparison to norm values (table 8). Participants with at least one present comorbid axis-I disorder (total group) showed significantly higher rates of somatization (T = 55,44 vs. T = 49,01; t = -3.10, p < .01) than participants without present comorbid Axis I disorders. In addition, significant higher rates for obsessive-compulsive symptoms, anxiety, hostility, phobic anxiety, paranoid ideation, psychoticism, global severity index and positive symptom total score were found in participants with one or more present comorbid mental disorders (p < .05).

**Table 8:** SCL-90-R scores

	Total Mean (SD)	No comorbidity Mean (SD)	Comorbidity^+^ Mean (SD)	t	p
Somatization	51.61 (12.56)	49.01 (11.89)	55.44 (12.63)	-3.1	.003
Obsessive-compulsive symptoms	52.74 (12.58)	50.61 (11.61)	55.88 (13.39)	-2.5	.014
Interpersonal sensitivity	50.34 (11.08)	49.65 (9.33)	51.35 (13.28)	-.80	.406
Depression	55.18 (11.67)	53.68 (10.80)	57.40 (12.61)	-1.9	.063
Anxiety	54.99 (10.73)	53.14(10.16)	57.70 (11.07)	-2.5	.013
Hostility	52.89 (10.85)	51.38 (10.50)	55.11 (11.07)	-2.0	.045
Phobic anxiety	52.48 (10.44)	50.77 (8.66)	54.98 (12.27)	-2.2	.028
Paranoid ideation	52.02 (11.06)	50.07 (9.40)	54.89 (12.68)	-2.5	.016
Psychoticism	53.05 (10.95)	51.49 (10.70)	55.35 (11.01)	-2.1	.039
Global severity index	53.66 (11.70)	51.74 (10.89)	56.49 (12.36)	-2.4	.017
Positive symptom distress index	57.86 (11.59)	56.35 (12.11)	60.09 (10.49)	-1.9	.060
Positive symptom total score	51.37 (10.98)	49.62 (10.37)	53.95 (11.43)	-2.3	.021

*Note: *^+^Comorbid diagnosis without ADHD & CD.

No relationship was found between substance-use clusters (as listed in table 3) and symptom distress scores.

### School attendance

106 patients (mean age = 16.05, SD = 1.09) still required mandatory schooling during the current school year upon admission. Of this subgroup, 33.0% had not at all attended school during the last two months prior to admission. The mean number of absent days for actually school attending participants (n = 71) during the last two months (46 days of school attendance) was 17.72 days (SD = 18.40). 51.4% of the school aged participants had been temporarily expelled from school at least once, 32.4% had to change schools as a disciplinary action. All participants were asked to rate their performance at school during the last year (or last year of school attendance in case of no current school attendance) on a three-point Likert scale ranging from below average (1) over average (2) to above average (3). 51.3% rated their school achievement below average, 45.3% average and 3.3% above average.

### Gender differences

Female participants suffered significantly more often from one or more lifetime and one or more present comorbid mental disorders (total group) (73.0% vs. 36.8%; phi = .312, p = .000 and 62.2% vs. 33.3%; phi = .253, p = .002, respectively). In detail, female participants significantly more often fulfilled criteria for lifetime and present PTSD (18.9% vs. 4.4%; phi = .231, p < .010), present (37.8% vs. 17.5%; phi = .209, p = .014) and lifetime (40.5% vs. 21.9%; phi = .181, p = .033) anxiety disorders, present (21.6% vs. 5.3%; phi = .243, p = .006) and lifetime (32.4% vs. 8.8%; phi = .288, p = .001) somatoform disorders than males. A female preponderance (diagnoses include ADHD and CD) was also detectable in the underage subgroup but did not reach statistical significance (present diagnosis: 66.7% vs. 48.0% vs.; phi = .169, p = .083; lifetime: 90.0% vs. 77.8%; phi = .145, p = .241). No significant difference in the mean number of comorbid diagnoses of patients with at least one comorbid disorder (without ADHD & CD) between females and males was found (present: 1.3 vs. 1.5, T = .85, p = .40; lifetime: 1.44 vs. 1.62, T = .76, p = .45). Additional t-tests showed no significant differences in symptom distress measured with SCL-90-R between male and female participants. Data from the subgroup (additionally evaluated for ADHD and CD) indicated no relationship between gender and rates of CD or ADHD (present and lifetime).

## Discussion

Due to the different forms of treatment, the evaluation of SUD prevalences in clinical samples is difficult. Nevertheless our results are basically consistent with other results [21,22,28]. In contrast to distributions of SUD found in studies on adolescents in the German general population [45], cannabis and amphetamine SUD seem to be overrepresented in our sample whereas alcohol related disorders were proportionally less often. Regarding the severity of abuse or dependence (our cannabis patients used this drug on average on 62% of the days of a month; Deas et al. [28] reported only half as many drugs for their cannabis users) and social deviances (data from the subgroup: 41.5% present comorbid conduct disorder), our sample represents a highly affected and deviant group of drug using adolescents.

Our SUD-patients most frequently suffered from comorbid mental disorders, predominantly conduct disorder and often anxiety and mood disorders. The high general risk of present comorbidity (40.5%; patients with additional K-SADS: 67.7%) found in this study is comparable to rates reported by most other studies (61% to 88%) of clinical SUD-samples [13]. In accordance to previous studies [22,25,30,31], our results affirm the high prevalence of comorbid disruptive behaviour symptoms in adolescent SUD-patients. High lifetime rates of CD have also been found by other authors [17]. In contrast to a some studies [14,25,29] our sample demonstrated comparatively moderate rates of ADHD which were similar to those reported by Wise et al. [26], Hannesdóttir et al. [23] and also by Grilo et al. [15] who found no difference in rates of ADHD between psychiatric inpatients with and without SUD.

In comparison with studies that assessed axis-I disorder rates in the German general population, our rates of lifetime diagnoses seem to be only slightly higher than rates found in representative cohorts: Essau et al. [3] scanned 1035 adolescents (aged 12 to 17) of the general population also using the German version of the CIDI and found somewhat lower rates (according to DSM-IV) for affective disorders (17.9% vs. 21.9%), anxiety disorders (18.6% vs. 26.5%, especially PTSD: 1.6% vs. 7.9%) and somatoform disorders (13.1% vs. 14.6%). With regard to the general lifetime occurrence of one or more axis-I disorders (including ADHD and CD), adolescents studied by Essau et al. [3] showed a substantially lower rate of psychiatric morbidity (Essau et al. s data includes also SUD) (41.9% vs. 81.5%). This difference can partially be explained by the high rate of conduct disorder in our sample. Another large (n = 3021) representative epidemiological study [2] also found lower rates of axis-I disorders in the general population; these participants (aged 14 to 24) less often fulfilled criteria for present axis-I disorders in general (without SUD, ADHD and CD) (17.5% vs. 40.5%), mood disorders (10.1% vs. 19.2%) especially dysthymic disorder (2.9% vs. 15.9%), anxiety disorders (9.3% vs. 22.5%) and somatoform disorders (0.7% vs. 9.3%) than participants from our sample which affirms the assumption of higher psychopathology in adolescents with SUD.

Except for the positive distress index in patients with at least one comorbid diagnosis, data obtained via the SCL-90-R demonstrated no clinically significant (T ≥ 60) degree of psychological distress either in patients with or without comorbidity. Considering the impairments which are most often associated with mental disorders, SUD and broken home situations, these results are difficult to interpret. Dissimulation to avoid long-term treatment, distorted self-perception and the relieving influence of inpatient treatment ("honeymoon effect") could possibly account for these results.

Comparable to Hovens et al. [29] (54% of the participants had dropped out of school) and Grella et al. [22] (38% not attending school), our data suggest that adolescent SUD is highly linked to school refusal and weak performance: In the two months prior to admission only 67.6% of the participants attended school or a comparable institution at least occasionally, being on average absent every other day. In possible relation to this behaviour, half of the participants judged their school performance as below average.

Our finding that more girls suffer from comorbid disorders than boys is consistent with the sparse literature [28,30] however some investigators did not find this relation [15]. Considering the different forms of treatment and study settings, this apparent inconsistency may reflect the effect of selective samples. The overrepresentation of boys (75.5%) in our clinical sample of SUD patients basically seems to reflect the proportion of substance abusing boys and girls in the German general population [2,45].

## Limitations

First of all, our sample is highly selective due to local modalities of admission. Transferences to other population groups are therefore difficult. In the light of the fact that substance use preferences and availability do vary across Germany and Europe, our- two-centre-design limits the generalisability of our results. However we provided data on days of substance use per month and school attendance to enable comparisons. Furthermore, our sites cover both an urban and a rural region, limiting the restriction on one possible sub-culture. At the present time, it is difficult to estimate the direction and impact of this possible bias. Incorrectly too high as well as too low rates of comorbidity are imaginable.

The sole implementation of the child version of the K-SADS-PL was inevitable (regarding the familiar difficulties the participants expressed) but led to a limited reliability of the diagnoses of CD and ADHD. Symptoms of external disorders (e.g. CD and ADHD) are underreported by adolescents in comparison to their parents [46]. It is impossible to judge to which extent some of the diagnosed disorders might not actually reflect a disorder directly attributable to the consequences of SUD, thus rendering the diagnosis of a substance induced disorder more appropriate.

## Conclusions

The high rate of comorbid psychopathology in inpatient SUD-patients, particularly conduct disorder has implications for therapy and framework of specialized treatment-units. Three-quarter of all patients show distinct comorbid psychopathology and SUD therapists should be able to take up this challenge. Patients with such a high rate of conduct disorder require specialised forms of treatment able to cope with high levels of aggression and treatment abortion often associated with CD.

Future research should investigate the causal and temporal relationship between conduct disorder and SUD, especially in respect of early developmental trajectories. Besides mental disorders, the high rate of school refusal and truancy should also be considered as important part of the substance use problem. Existing school refusal treatment programmes should be aware of the high co-occurrence whereas SUD-treatment units should carefully evaluate psychological causes of school refusal and emphasize school reintegration. Finally, controlled longitudinal comparative studies are needed to test the possible positive effect of comorbidity-considering treatment programmes.

## Competing interests

The authors declare that they have no competing interests.

## Authors' contributions

Authors TL, NQ, NS, PM and JH designed the study and wrote the protocol. TL and NS conducted literature searches and provided summaries of previous research studies. TL conducted the statistical analysis. TL, AS, EO and GW conducted the assessment of the participants. TL and JH wrote the manuscript and all authors contributed to and have approved the final manuscript.

All authors have read and approved the final manuscript.
